# Deep Learning–Based Detection of Early Renal Function Impairment Using Retinal Fundus Images: Model Development and Validation

**DOI:** 10.2196/23472

**Published:** 2020-11-26

**Authors:** Eugene Yu-Chuan Kang, Yi-Ting Hsieh, Chien-Hung Li, Yi-Jin Huang, Chang-Fu Kuo, Je-Ho Kang, Kuan-Jen Chen, Chi-Chun Lai, Wei-Chi Wu, Yih-Shiou Hwang

**Affiliations:** 1 Department of Ophthalmology Chang Gung Memorial Hospital, Linkou Medical Center Taoyuan Taiwan; 2 College of Medicine Chang Gung University Taoyuan Taiwan; 3 Department of Ophthalmology National Taiwan University Hospital Taipei Taiwan; 4 Acer Healthcare Incorporated New Taipei Taiwan; 5 Center for Artificial Intelligence in Medicine Chang Gung Memorial Hospital, Linkou Medical Center Taoyuan Taiwan; 6 Department of Nephrology Yang Ming Hospital Taoyuan Taiwan

**Keywords:** deep learning, renal function, retinal fundus image, diabetes, renal, kidney, retinal, eye, imaging, impairment, detection, development, validation, model

## Abstract

**Background:**

Retinal imaging has been applied for detecting eye diseases and cardiovascular risks using deep learning–based methods. Furthermore, retinal microvascular and structural changes were found in renal function impairments. However, a deep learning–based method using retinal images for detecting early renal function impairment has not yet been well studied.

**Objective:**

This study aimed to develop and evaluate a deep learning model for detecting early renal function impairment using retinal fundus images.

**Methods:**

This retrospective study enrolled patients who underwent renal function tests with color fundus images captured at any time between January 1, 2001, and August 31, 2019. A deep learning model was constructed to detect impaired renal function from the images. Early renal function impairment was defined as estimated glomerular filtration rate <90 mL/min/1.73 m^2^. Model performance was evaluated with respect to the receiver operating characteristic curve and area under the curve (AUC).

**Results:**

In total, 25,706 retinal fundus images were obtained from 6212 patients for the study period. The images were divided at an 8:1:1 ratio. The training, validation, and testing data sets respectively contained 20,787, 2189, and 2730 images from 4970, 621, and 621 patients. There were 10,686 and 15,020 images determined to indicate normal and impaired renal function, respectively. The AUC of the model was 0.81 in the overall population. In subgroups stratified by serum hemoglobin A_1c_ (HbA_1c_) level, the AUCs were 0.81, 0.84, 0.85, and 0.87 for the HbA_1c_ levels of ≤6.5%, >6.5%, >7.5%, and >10%, respectively.

**Conclusions:**

The deep learning model in this study enables the detection of early renal function impairment using retinal fundus images. The model was more accurate for patients with elevated serum HbA_1c_ levels.

## Introduction

### Background

Chronic kidney disease (CKD) is defined as a gradual loss of renal function, and it can progress to an advanced stage, termed end-stage renal disease (ESRD). According to the 2016 annual report of the US Renal Data System [1], the incidence of treated ESRD increased gradually at the rate of 2%-4% from 2003 to 2016 in almost one-third of all countries [1]. Taiwan, in particular, had the highest incidence of treated ESRD (493 patients per million in the general population) and the highest prevalence of treated ESRD (3392 patients per million in the general population) among all countries worldwide [1]. According to Taiwan’s National Health Insurance 2018 report [2], CKD incurred the highest medical costs in the country, approximately US $1.7 billion. Therefore, progress is required in the prevention and screening of kidney disease in Taiwan. In all the etiologies of CKD, diabetes is a leading cause; it has been estimated that 1 in 4 adults with diabetes have impaired renal function [3]. Therefore, the monitoring of renal function is especially important for patients with diabetes; it is also crucial in countries where ESRD is prevalent.

With the increasing sophistication of artificial intelligence, deep learning has been increasingly applied to various types of medical imaging analysis, especially ophthalmology imaging [4]. Among ophthalmology imaging techniques, retinal imaging has been used to establish deep learning models for detecting not only eye diseases (eg, diabetic retinopathy and glaucoma) [5,6] but also systemic cardiovascular risks [7]. The microvascular network in the retina can be easily observed; it is structurally and physiologically similar to the vascular structures of many other systems or organs and can be used in the evaluation of various disorders, including systemic hypertension, coronary artery disease, and central nervous disorders [8-10]. Studies have also demonstrated that changes in the retinal vasculature are associated with renal dysfunction and reduced estimated glomerular filtration rate (eGFR) [11,12].

### Objective

Scholars have recommended applying artificial intelligence to the management and prevention of kidney disease [13]. However, few studies have developed deep learning–based methods for detecting early renal function impairment from retinal images. Therefore, we established a deep learning model to detect early renal function impairment from retinal fundus images. We also evaluated the performance of our model when applied to patients with diabetes.

## Methods

### Study Population

In this retrospective study, we included patients who underwent retinal fundus imaging examinations and laboratory tests at any time between January 1, 2001, and August 31, 2019, at Chang Gung Memorial Hospital (CGMH), Linkou Medical Center, Taoyuan, Taiwan. The retinal fundus images were taken with fundus cameras (Topcon Medical Systems, KOWA, and Digital Non-Mydriatic Retinal Camera, Canon). The laboratory tests conducted for serum creatinine and serum hemoglobin A_1c_ (HbA_1c_) were respectively performed with a colorimetric method and high-performance liquid chromatography at the CGMH Department of Laboratory Medicine. Demographic data, including those on age and sex, were also retrieved from CGMH’s electronic medical record system. This study was approved by the CGMH Institutional Review Board (CGMH IRB No. 201901544B0), and the requirement for informed consent was waived because patient data were deidentified. The study was conducted in accordance with the Declaration of Helsinki.

### Data Management

After the data were retrieved, retinal fundus images were linked to the corresponding renal functions, which were measured by eGFR. In our study, the eGFR was calculated using the Modification of Diet in Renal Disease (MDRD) equation, which includes the patient’s age, sex, and serum creatinine, as revised by Levey et al [14]. We defined early renal function impairment as eGFR <90 mL/min/1.73 m^2^, which was equal to or more severe than the mildly decreased glomerular filtration rate according to the definition published in the 2012 guidelines of “Kidney Disease: Improving Global Outcomes” [15]. We only included laboratory tests that had been conducted within 3 months before or after the corresponding retinal fundus images were captured. Patients without available serum creatinine results were excluded. We deidentified the data after the images and laboratory data were linked. Subsequently, we excluded retinal fundus images that had color filters, were merged, or were neither macula- nor disk-centered. For an image to be included, both the macula and disk were required to be visible. We also excluded poor-quality images, such as those that had a low resolution, were out of focus, had a large halo, or had a large shadow. Multimedia Appendix 1 presents some examples of the excluded images*.*

### Model Architecture

The model architecture is illustrated in Figure 1. To reduce the variation of illumination and camera resolution between the different retinal images, all images were processed using the method proposed by Graham [16]. All images were resized to a resolution of 224 × 224 × 3 and were processed to reduce variance in illumination between images before running the algorithm. For the convolutional neural network (CNN), we selected VGG-19 formulated by the Visual Geometry Group [17]. We selected VGG-19 because it exhibited the best performance in our preliminary model training relative to ResNet, Inception V3, and Inception V4. Furthermore, in previous research, VGG-19 exhibited comparable performance to other deeper CNNs in medical imaging analysis in general and in ophthalmological imaging in particular [18]. After the CNN retrieved the image feature, a batch normalization layer was added to accelerate training, and the features were flattened to 1-dimensional vectors. Subsequently, we added 3 fully connected layers that had a nonlinear rectified linear unit (ReLU) activation function and 1 final output layer with the softmax activation function. The results were classified results into 2 classes—class 0 and 1, which represented normal and impaired renal function, respectively. The probability for each class was presented. As presented in Figure 1, the probability of disease was 0.76, and a saliency map was generated based on the features marked as determinative for the detection of renal function impairment.

**Figure:**
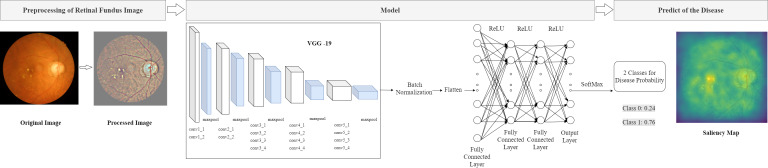
Architecture of the model for detecting early renal function impairment from retinal fundus images. ReLU: rectified linear unit.

### Model Training and Performance

The data sets of all patients were partitioned into nonoverlapping training, validation, and testing sets at an 8:1:1 ratio, and the images from each patient were linked to the corresponding renal function results. The model was trained, validated, and tested on the basis of the images. The model was trained on a workstation with an Intel Xeon Silver 4110 CPU at 2.10 GHz, a NVIDIA GeForce GTX 1080 Ti (with 11 GB of video memory) graphics card, and 125 GB of RAM. For this model, the learning rate and batch size were set as 0.000005 and 32, respectively. An Adam optimizer was used, and the model was trained up to 120 epochs. The model was established based on the achievement of maximum accuracy and minimum loss in the validation set. The learning curve of the model is presented in Multimedia Appendix 2. To analyze the model prediction, we generated saliency maps (Figure 1), which identified the region of the retinal fundus photo that contributed to the model’s determination of renal function impairment. We also classified the testing set according to the patient’s HbA_1c_ levels. Furthermore, the model performance was evaluated at HbA_1c_ levels of ≤6.5%, >6.5%, >7.5%, and >10.0% in the testing data set.

### Statistical Analysis

For the demographic data, continuous variables were expressed in terms of the mean (SD). Chi-square tests and *t* tests were conducted for descriptive analyses of categorical (sex) and continuous (age and HbA_1c_) variables, respectively. To analyze the performance of our model, receiver operating characteristic (ROC) curves were plotted, and the area under the curve (AUC) for each ROC curve was calculated. AUC values of 0.7-0.8 and >0.8 indicated acceptable discrimination and excellent discrimination, respectively. An AUC value of 1 represented perfect discrimination, and AUC value of 0.5 represented no or random discrimination [19]. We also measured the sensitivity, specificity, positive predictive value (PPV), and accuracy of the model. Model performance was evaluated using the images in the testing set. Statistical significance was indicated if *P*<.05. Statistical analyses were conducted using SPSS (Version 23, IBM Corp).

## Results

### Demographic Characteristics

In this study, we initially included 7167 patients with 51,666 retinal fundus images. We then excluded 13.32% (955/7167) patients and 50.24% (25,960/51,666) images after applying the exclusion criteria. The remaining 25,706 retinal fundus images from 6212 patients were included in the final analysis, and each patient may have a different number of images. The variance was 1 to 33 images per patient. The training, validation, and testing sets comprised 20,787, 2189, and 2730 images from 4970, 621, and 621 patients, respectively (Table 1).

**Table 1:** Distribution of patients with clinical information in the training, validation, and testing groups.

Characteristic		Total (N=6212)	Training (n=4970)	Validation (n=621)	Testing (n=621)
**Sex, n (%)**					
	Male	3363 (54.14)	2689 (54.10)	339 (54.6)	335 (53.9)
	Female	2849 (45.86)	2281 (45.90)	282 (45.4)	286 (46.1)
Age (years), mean (SD)		57.6 (16.6)	58.7 (15.9)	51.0 (19.1)	51.6 (17.4)
eGFR^a^ (ml/min/1.73 m^2^), mean (SD)		78.6 (32.6)	77.8 (32.2)	86.5 (34.1)	80.4 (35.6)
HbA_1c_^b^ (%), mean (SD)		7.6 (2.0)	7.6 (1.9)	7.6 (1.8)	7.9 (2.1)

^a^eGFR: estimated glomerular filtration rate.

^b^HbA_1c_: hemoglobin A_1c_.

Each patient was randomly assigned to a group, and all images from a patient belonged only to the group the patient was assigned to. With regard to demographic characteristics, 54.14% (3363/6212) of the patients were male, and the mean age of all patients was 57.6 (SD 16.6) years. As for clinical characteristics, the mean eGFR and serum HbA_1c_ levels were 78.6 mL/min/1.73 m^2^ (SD 32.6) and 7.6% (SD 2.0%), respectively. Table 2 presents the clinical information for normal and impaired renal function (eGFR <90 mL/min/1.73 m^2^) in our study population.

**Table 2:** Clinical information of patients with normal or impaired renal function (N=6212); all *P* values are <.001.

Characteristic		Normal renal function (n=3108)	Impaired renal function (n=3104)
**Sex, n (%)**			
	Male	1539 (49.52)	1824 (58.76)
	Female	1569 (50.48)	1280 (41.24)
Age (years), mean (SD)		47.2 (16.1)	64.1 (13.1)
HbA_1c_^a^ (%), mean (SD)		7.7 (2.1)	7.5 (1.9)

^a^HbA_1c_: hemoglobin A_1c_.

Compared with patients with healthy renal function, patients with impaired renal function were more likely to be male (impaired vs healthy: 58.3% vs 49.1%; *P*<.001), older adults (64.1 years vs 47.2 years; *P*<.001), and with a lower serum HbA_1c_ level (7.5% vs 7.7%; *P*<.001). Multimedia Appendix 3 shows the clinical information in patients with stratified HbA_1c_ levels in the testing set.

### Model Performance

**Figure 2:**
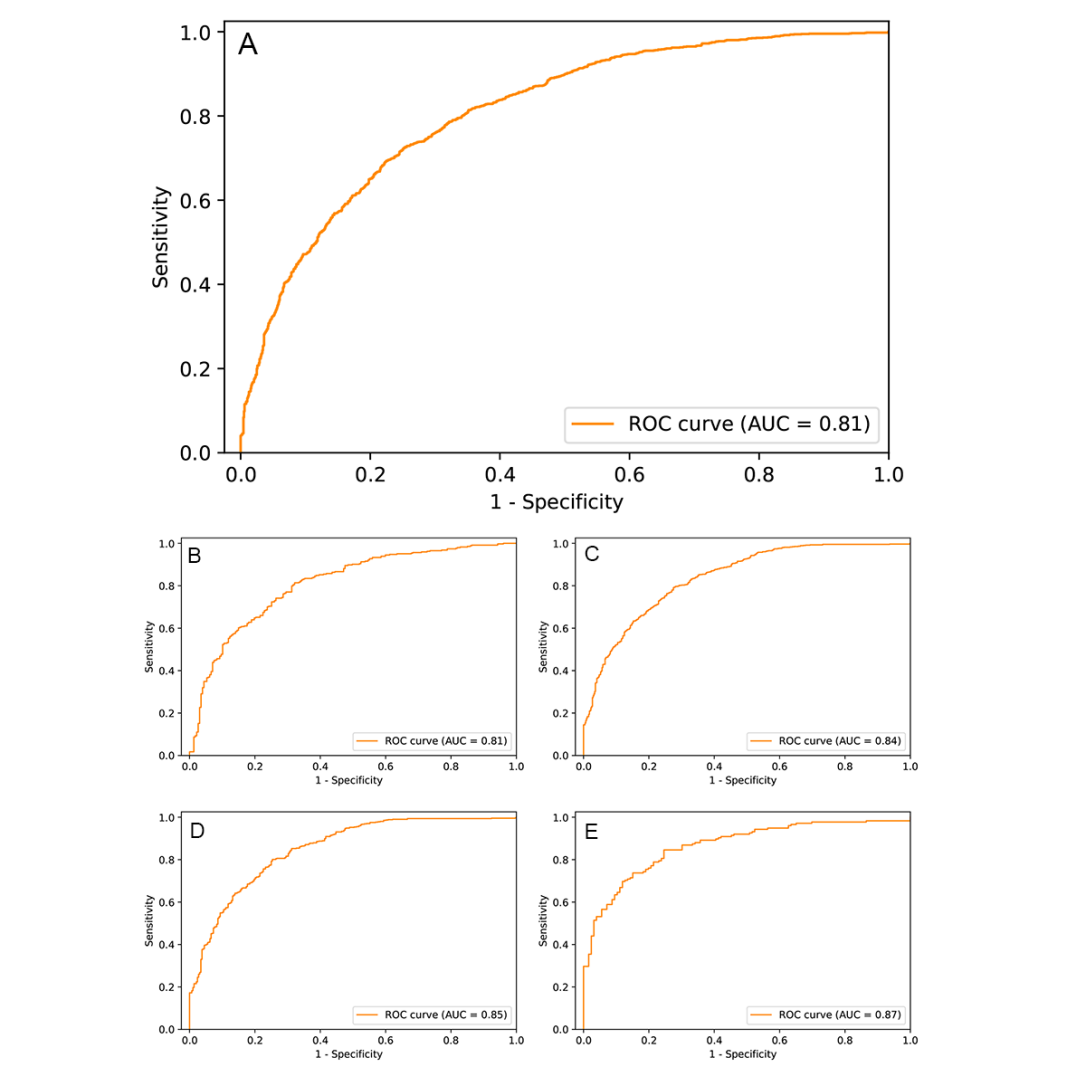
ROC curves for the model in detecting early renal function impairment in different groups of patients. ROC curves for (A) all patients (AUC = 0.81, sensitivity = 0.83, specificity = 0.62, PPV = 0.73, accuracy = 0.73); (B) patients with HbA_1c_ ≤ 6.5% (AUC = 0.81, sensitivity = 0.84, specificity = 0.62, PPV = 0.77, accuracy = 0.75), (C) patients with HbA_1c_ > 6.5% (AUC = 0.84, sensitivity = 0.89, specificity = 0.61, PPV = 0.77, accuracy = 0.77), (D) patients with HbA_1c_ > 7.5% (AUC = 0.85, sensitivity = 0.89, specificity = 0.60, PPV = 0.82, accuracy = 0.79), and (E) patients with HbA_1c_ > 10.0% (AUC = 0.87, sensitivity = 0.89, specificity = 0.61, PPV = 0.77, accuracy = 0.77). AUC: area under the curve; HbA_1c_: hemoglobin A_1c_; PPV: positive predictive value; ROC: receiver operating characteristic.

The ROC curves obtained from tests of our model are presented in Figure 2. Model performance for subgroups stratified by serum HbA_1c_ level was also tested. Model performance increased gradually with serum HbA_1c_ level.

### Saliency Maps

**Figure 3:**
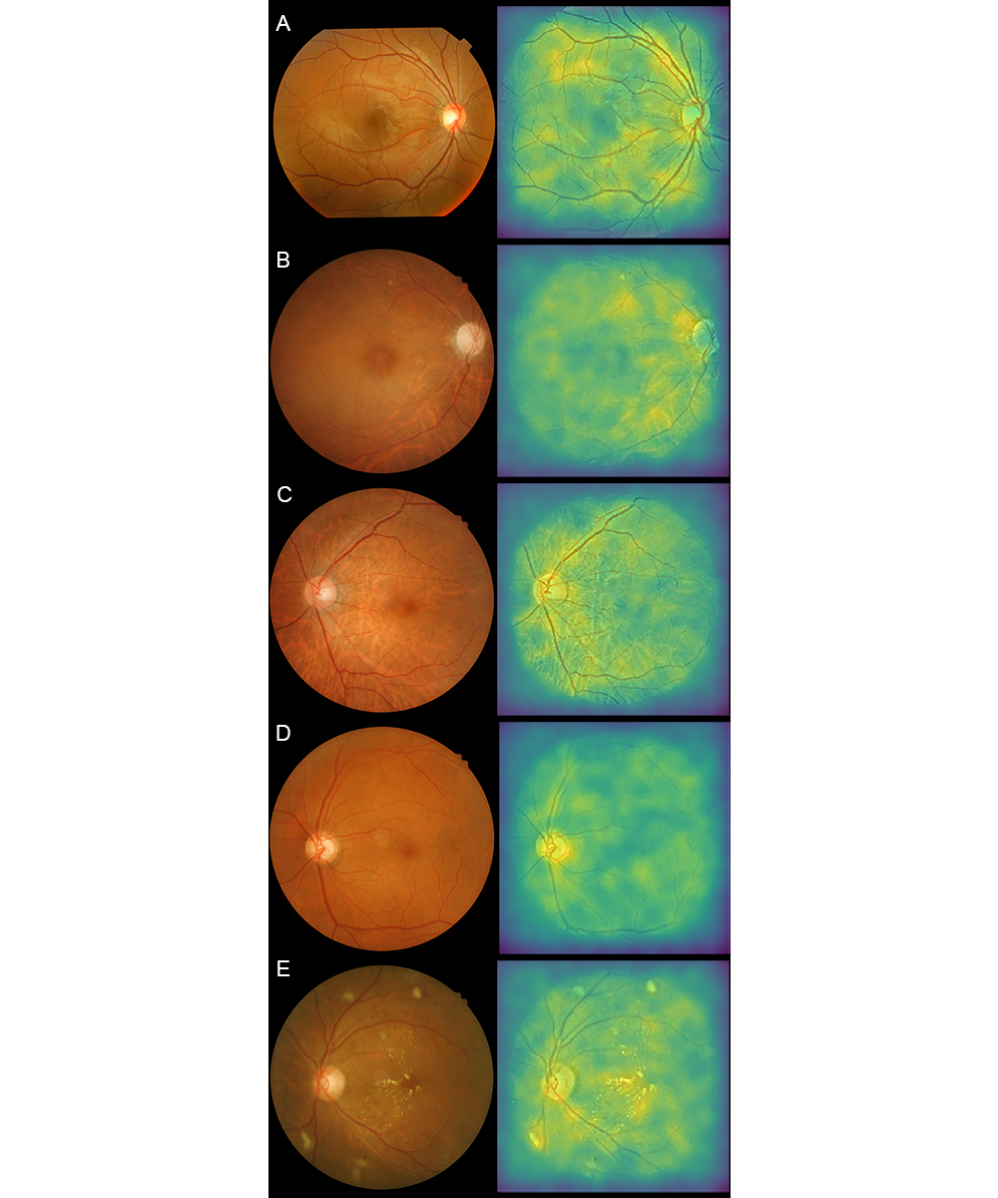
Selected retinal fundus images and their corresponding saliency maps in true-negative and true-positive cases. (A) No renal function impairment detected. Patient’s eGFR = 102.6 mL/min/1.73 m^2^ and HbA_1c_ = 13.4%. (B) Renal function impairment detected. Patient’s eGFR = 40.0 mL/min/1.73 m^2^ and HbA_1c_ = 5.1%. (C) Renal function impairment detected. Patient’s eGFR = 50 mL/min/1.73 m^2^ and HbA_1c_ = 6.5%. (D) Renal function impairment detected. Patient’s eGFR = 80.5 mL/min/1.73 m^2^ and HbA_1c_ = 7.3%. (E) Renal function impairment detected. Patient’s eGFR = 67.7 ml/min/1.73 m^2^ and HbA_1c_ = 8.9%. eGFR: estimated glomerular filtration rate; HbA_1c_: hemoglobin A_1c_.

Representative saliency maps are presented in Figure 3, where the regions responsible for the prediction of impaired renal function are highlighted in the lighter color. In Figure 3, the retinal-vessel features are marked for a true-positive case with a relatively normal retinal fundus image. Common signs of retina abnormality, such as exudation, hemorrhage, and drusen, also played a role in the detection of renal function impairment.

## Discussion

### Main Findings

In this study, we developed a deep learning model for detecting early renal function impairment from retinal fundus images. The AUC of the model was 0.81 for the detection of early renal function impairment in the general population, and the model performed better when applied to patients with diabetes or patients with elevated serum HbA_1c_ levels.

### Importance of Renal Function Screening

The 2016 annual report of the US Renal Data System [1] notes that ESRD is becoming increasingly prevalent in many countries, underscoring the increased burdens of CKD and ESRD on society. Taiwan has a high incidence and prevalence of CKD and ESRD, and the country bears significant health care burden associated with CKD and ESRD [1,2]. Thus, several studies in Taiwan have evaluated the etiology and screening of kidney diseases [20,21]. In Taiwan, CKD prevention has been hampered by low public awareness, infrequent eGFR measurements, and delayed referrals [22,23]. Although a study suggested the importance of comprehensive renal function screening in high-risk populations, such as patients with diabetes [15], evidence for the cost-effectiveness and benefits of routine screening for CKD remain inconclusive because commonly used tests with urine or blood are inconvenient and invasive [24].

### Deep Learning in Renal Function Using Ultrasonography

Deep learning methods provide a potential solution to this problem. With the increasing sophistication of artificial intelligence, deep learning has been increasingly applied in various fields, including medicine [25]. The use of artificial intelligence for management of kidney disease has been recently proposed, and its potential has been well recognized by physicians [13]. Kuo et al [26] developed a deep learning model for predicting renal function by using kidney ultrasound images. Their model was more accurate (0.86) in detecting cases with eGFR <60 mL/min/1.73 m^2^ than the judgments of experienced nephrologists (0.60-0.80). Although our model had lower overall accuracy (0.73 for all patients and 0.79 for patients with HbA_1c_ > 7.5%) relative to theirs, our model’s accuracy is still comparable with that of the judgments of experienced nephrologists employing ultrasound images. Moreover, our model could detect early renal function impairment with eGFR <90 mL/min/1.73 m^2^, a functionality that was not evaluated by Kuo et al [26].

### Deep Learning Using Retinal Fundus Images

Retinal fundus imaging can be executed even by untrained medical staff and has high accessibility. Furthermore, a patient’s retinal fundus images can be captured in less than 10 minutes, and the patient can be promptly referred to a specialist if a problem is detected [27]. A previous review on deep learning in ophthalmology noted that retinal fundus images can be used to identify several eye diseases, including glaucoma, macular degeneration, refractive errors, and, most importantly, diabetic retinopathy [18]. Furthermore, systemic cardiovascular risks can also be determined from retinal images [7]. Those results suggest the potential of using retinal photography for large-scale disease screening.

### Using Retinal Fundus Images for Renal Function Prediction

In our study, we developed a deep learning model to detect early renal function impairment. The model had excellent discrimination (AUC=0.81; excellent discrimination was defined as AUC >0.8) [19]. The saliency maps revealed that features in retinal vasculature and of hemorrhages and exudations were influential in the determination of impaired renal function. This finding is compatible with the findings of previous reports on specific retinal microvascular and structural changes in renal function impairment [11,28]. When applied to patients with diabetes, our model had a sensitivity as high as 0.89 but a specificity of only 0.60. We noted that our model produced several false positives for patients who shared some similar ophthalmic pathologies presenting on the fundus images. These pathologies included subretinal fluid, optic disc swelling caused by optic neuritis, and retinal scarring (Multimedia Appendix 4). However, no robust association between these pathologies and renal function is indicated in the literature. As noted in the saliency maps, the model identified retinal vessel characteristics and the presence of hemorrhage and exudation. Subretinal fluid and optic disc swelling may alter retinal vascular features and thus affect the model prediction. Ocular infection or inflammation was also presented with retinal vascular change, hemorrhage, exudation, and pigmented scars [29], which may be similar to the retinal presentation of impaired renal function. Therefore, these coexisting ocular pathologies may have reduced model specificity. For future studies on deep learning, we suggest the use of multimodal retinal images to predict renal function impairment; the analysis of multimodal retinal images has been reported to yield greater accuracy in diagnosing age-related macular degeneration [30].

### Comparison of Model Performance in Diabetes and Between the Previous Study

Our model had a greater AUC and sensitivity for higher HbA_1c_ levels (up to AUC=0.87 for HbA_1c_ >10%). Some possible explanations for this performance include more profound microvascular damage in patients with worse glucose control and the coexistence of signs of diabetic retinopathy and diabetic nephropathy, which were noted to be significantly associated [31,32]. A deep learning algorithm was recently formulated by a research group at the Singapore National Eye Center (SNEC) [33]. Their algorithm was used to detect CKD with eGFR <60 mL/min/1.73 m^2^ by using both retinal images and risk factors, individually and in combination, in 3 population-based screening databases from Singapore and China [33]. Their image-based model had an AUC of 0.91 in their internal validation (Singapore Epidemiology of Eye Diseases database), AUCs of 0.73 and 0.84 in their external testing (Singapore Prospective Study Program and Beijing Eye Study, respectively), and an AUC of 0.89 when applied in patients with diabetes. The overall performance of our model (AUC=0.81) is in between the performance levels of their model in their internal validation and external testing. This difference in performance is attributable to differences in patient characteristics or model architecture. Our hospital is a referral medical center with comprehensive ophthalmology equipment for the management of advanced eye diseases [34]. Compared with population-based screening databases, our database featured more patients with pathologies on the retina or other parts of the eye, which may have increased the likelihood of model misdiagnosis [35]. In addition, the model was trained using images from 1 of 3 types of fundus cameras and 1 of 2 different image formats (JPEG or PNG). This variety likely affected the predictive performance of the model. Specifically, when our model was applied to the subgroup of patients with diabetes, its performance (AUC = 0.84 in HbA_1c_ >6.5%, 0.85 in HbA_1c_ >7.5%, and 0.87 in HbA_1c_ >10.0%) was comparable to that of the SNEC model.

### Study Limitations

Our study has some limitations. First, the results of the MDRD formula for calculating eGFR did not reflect definite renal function; variations related to ethnicity have been reported, and this measure was noted to be less accurate when applied to the Taiwanese population [21,36]. Second, as we aimed to detect early renal function impairment (ie, eGFR <90 mL/min/1.73 m^2^), we did not test the efficacy of our model in predicting advanced kidney diseases. Third, we discarded poor-quality fundus images before training the model. However, poor-quality images are encountered in clinical settings, and model performance may thus be affected by factors such as patient cooperation and medial opacities of the eye and small pupils [27]. Although retinal fundus imaging is a relatively accessible test, the feasibility of our model in real-world applications requires further investigation. Fourth, the model’s detection of renal function may be affected by signs from some ocular diseases that are related to neither systemic vascular function nor renal function. For example, certain retinal infections may alter the model’s prediction of renal function impairment; such infections are not associated with systemic vascular function but share a common feature, namely the presence of hemorrhages or exudates on the retina. By contrast, renal function impairment with nonvascular causes, such as urinary tract obstruction, may not present vasculature or retinal abnormality in fundus images during the early disease phase. In our study, selection bias may have occurred in the subpopulation with a referral medical center. This subpopulation has a higher proportion of patients with ocular diseases coexisting with other organic diseases. Fifth, we did not perform patient-matching between the training, validation, and testing groups. Thus, differences in clinical characteristics may have affected the learning and performance of the model. Finally, the function of this model lies in screening rather than diagnosis. A thorough kidney examination that includes ultrasonography and insulin clearance remains crucial.

### Conclusion

In conclusion, our study formulated and evaluated a deep learning model for predicting early renal function impairment. Our model also performed better, as indicated by the increased AUC, when applied to patients with diabetes or patients with elevated serum HbA_1c_ levels. Color fundus images are easy to obtain and can thus be feasibly applied to the detection of early renal function impairment, especially in patients with diabetes, in conjunction with our model.

## Appendix

Multimedia Appendix 1 Excluded retinal fundus images due to (a) the use of a color filter, (b) the image not being centered on the macula or disc, (c) blurriness, (d) the presence of glares, (e) poor light exposure, and (f) an invisible macula.

Multimedia Appendix 2 Learning curves of the model, demonstrating the learning rate in the epochs versus (a) train loss and (b) accuracy. The epoch of 56 with the lowest validation loss was selected for testing.

Multimedia Appendix 3 Clinical information of patients with normal or impaired renal function stratified by different HbA1c levels in the testing set.

Multimedia Appendix 4 Retinal fundus images from false-positive cases showing (a) a swollen optic disc due to idiopathic optic neuritis, (b) a chorioretinal scar caused by previous inflammation, and (c) subretinal fluid caused by retinal detachment.

## Abbreviations

CGMH: Chang Gung Memorial Hospital
CKD: chronic kidney disease
eGFR: estimated glomerular filtration rate
ESRD: end-stage renal disease
HbA_1c_: hemoglobin A_1c_
MDRD: Modification of Diet in Renal Disease
CNN: convolutional neural network
ReLU: rectified linear unit
ROC: receiver operating characteristic
AUC: area under the curve
PPV: positive predictive values
SNEC: Singapore National Eye Center
