# Supplementary figures and images for: Deep Learning–Based Detection of Early Renal Function Impairment Using Retinal Fundus Images: Model Development and Validation
Source: JMIR Med Inform. 2020 Nov 26;8(11):e23472. doi: 10.2196/23472 (PMC7728538; doi:10.2196/23472)

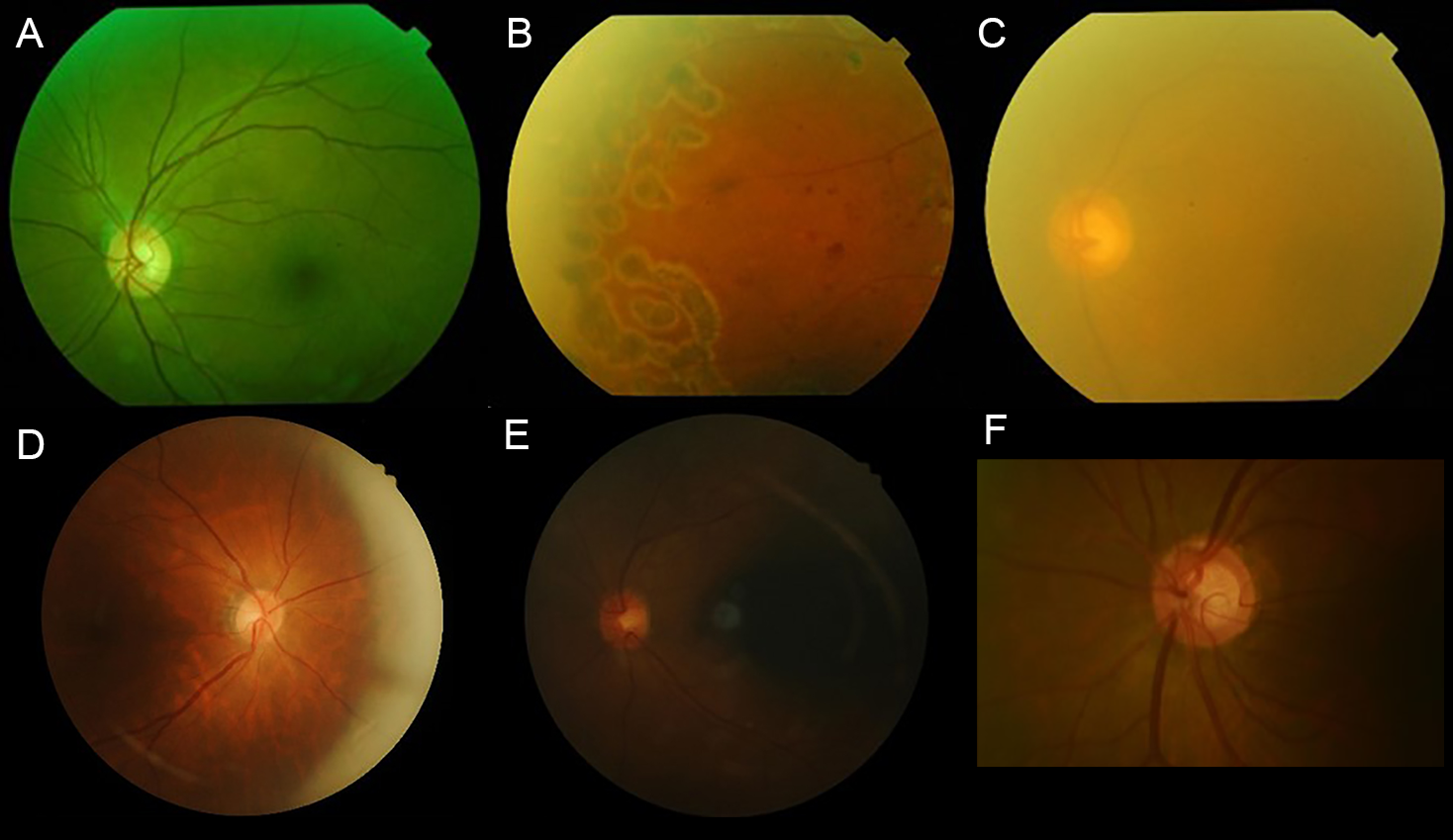

Supplement: Multimedia Appendix 1 [file medinform_v8i11e23472_app1.png]

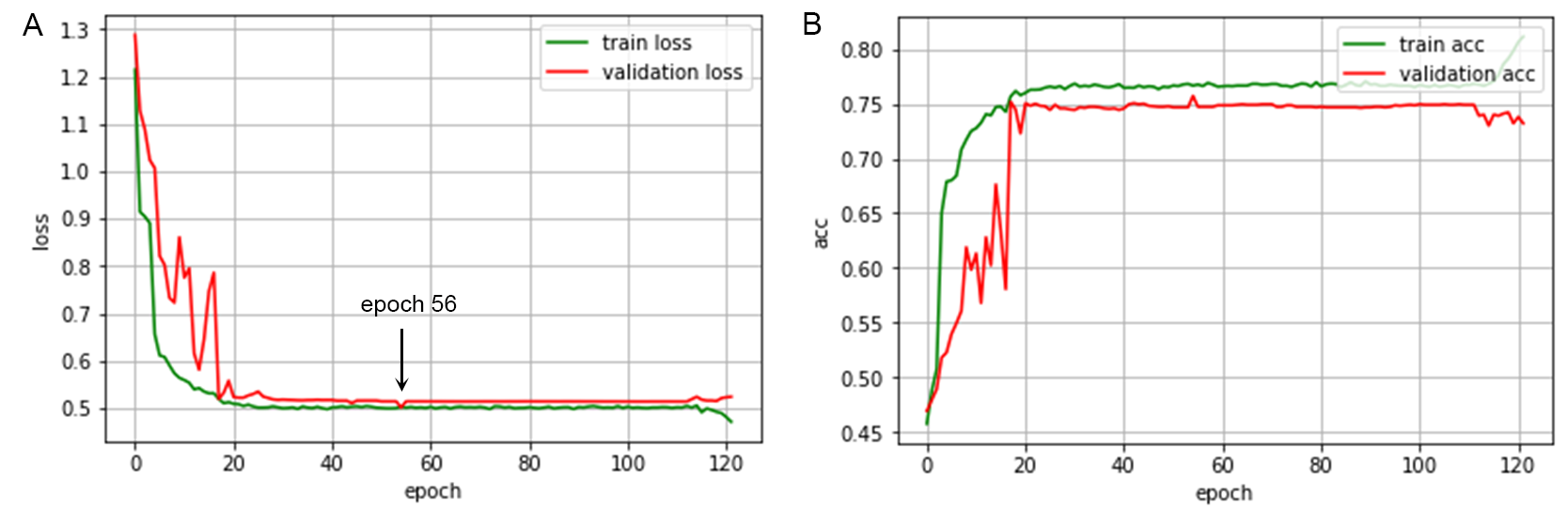

Supplement: Multimedia Appendix 2 [file medinform_v8i11e23472_app2.png]

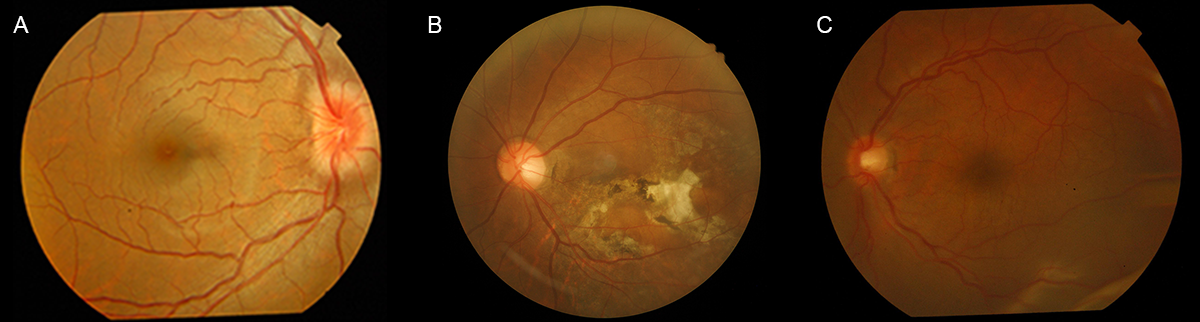

Supplement: Multimedia Appendix 4 [file medinform_v8i11e23472_app4.png]
