# Supplementary material for: Deep Learning–Based Detection of Early Renal Function Impairment Using Retinal Fundus Images: Model Development and Validation
Source: JMIR Med Inform. 2020 Nov 26;8(11):e23472. doi: 10.2196/23472 (PMC7728538; doi:10.2196/23472)
Supplement: Multimedia Appendix 3 [file medinform_v8i11e23472_app3.docx]

| **Supplementary Table 1.** Clinical information of patients with normal or impaired renal function stratified by different HbA1c levels in the testing set. | | | |
| --- | --- | --- | --- |
| Characteristics | Normal renal function | Impaired renal function | *P*-value |
| HbA1c < 6.5%, n | 49 | 60 |  |
| Male, n | 20 | 41 | .004 |
| Age, years^a^ | 47.7±13.7 | 62.0±12.4 | <.001 |
| HbA1c, %^a^ | 5.8±0.4 | 5.8±0.4 | .94 |
| HbA1c > 6.5% | 120 | 145 |  |
| Male, n | 68 | 76 | .13 |
| Age, years^a^ | 42.3±16.7 | 60.2±12.4 | <.001 |
| HbA1c, %^a^ | 8.9±2.4 | 8.6±1.8 | .009 |
| HbA1c > 7.5% | 76 | 107 |  |
| Male, n | 42 | 52 | .36 |
| Age, years^a^ | 39.2±16.5 | 59.1±12.7 | <.001 |
| HbA1c, %^a^ | 10.1±2.3 | 9.4±1.7 | <.001 |
| HbA1c > 10.0% | 27 | 33 |  |
| Male, n | 17 | 16 | .27 |
| Age, years^a^ | 38.1±15.2 | 53.7±16.2 | <.001 |
| HbA1c, %^a^ | 12.7±1.9 | 11.9±1.5 | .0014 |
| ^a^Continuous variables are presented as the mean ± standard deviation  n: number, HbA1c: hemoglobin A1c | | | |
